# Pharmacological Potential of Cilostazol for Alzheimer’s Disease

**DOI:** 10.3389/fphar.2019.00559

**Published:** 2019-05-22

**Authors:** Kenjiro Ono, Mayumi Tsuji

**Affiliations:** ^1^ Division of Neurology, Department of Medicine, Showa University School of Medicine, Tokyo, Japan; ^2^ Department of Pharmacology, Showa University School of Medicine, Tokyo, Japan

**Keywords:** Alzheimer’s disease, amyloid β-protein, oligomer, cilostazol, neurotoxicity

## Abstract

Alzheimer’s disease (AD), a slow progressive form of dementia, is clinically characterized by cognitive dysfunction and memory impairment and neuropathologically characterized by the accumulation of extracellular plaques containing amyloid β-protein (Aβ) and neurofibrillary tangles containing tau in the brain, with neuronal degeneration and high level of oxidative stress. The current treatments for AD, e.g., acetylcholinesterase inhibitors (AChEIs), have efficacies limited to symptom improvement. Although there are various approaches to the disease modifying therapies of AD, none of them can be used alone for actual treatment, and combination therapy may be needed for amelioration of the progression. There are reports that cilostazol (CSZ) suppressed cognitive decline progression in patients with mild cognitive impairment or stable AD receiving AChEIs. Previously, we showed that CSZ suppressed Aβ-induced neurotoxicity in SH-SY5Y cells *via* coincident inhibition of oxidative stress, as demonstrated by reduced activity of nicotinamide adenine dinucleotide phosphate oxidase, accumulation of reactive oxygen species, and signaling of mitogen-activated protein kinase. CSZ also rescued cognitive impairment and promoted soluble Aβ clearance in a mouse model of cerebral amyloid angiopathy. Mature Aβ fibrils have long been considered the primary neurodegenerative factors in AD; however, recent evidence indicates soluble oligomers to initiate the neuronal and synaptic dysfunction related to AD and other protein-misfolding diseases. Further underscoring the potential of CSZ for AD treatment, we recently described the inhibitory effects of CSZ on Aβ oligomerization and aggregation *in vitro*. In this review, we discuss the possibility of CSZ as a potential disease-modifying therapy for the prevention or delay of AD.

## Introduction

Alzheimer’s disease (AD), a progressive neurodegenerative disease, is associated with dementia. The brains of patients with AD are characterized by the occurrence of plaques primarily composed of amyloid β-protein (Aβ) and neurofibrillary tangles composed of tau protein (Selkoe and Hardy, 2016; Gao et al., 2018). Despite the recent advances in symptomatic therapy involving the use of N-methyl-D-aspartate receptor (NMDAR) antagonist and cholinergic drugs, no disease-modifying therapies (DMTs) exist, which directly ameliorate AD-related neurodegenerative processes at the present (Cummings et al., 2016).

Aβ aggregation is considered one of the most important pathogenic processes, i.e., the amyloid hypothesis; therefore, studies on DMTs have primarily focused on the agents that prevent the accumulation of tau deposits and Aβ in the central nervous system (Cummings et al., 2016). Indeed, *in vitro* and cell studies, human genetics analyses, and neurophysiological studies in animal models strongly implicate Aβ aggregation in AD-associated neurodegeneration *via* the promotion of oxidative stress, inflammation, and apoptosis (Selkoe and Hardy, 2016).

Aβ molecules aggregate to form soluble oligomers and fibrils (Ono, 2018). Subsequently, Aβ aggregates can directly cause neurodegeneration by acting on neurons or indirectly cause it by activating astrocytes and microglia, thereby triggering cytotoxic inflammatory cascades. Hence, to date, several DMTs have been developed targeting different Aβ aggregates (Ono, 2018).

Cilostazol (CSZ) is a selective phosphodiesterase (PDE) 3 inhibitor, which increases intracellular cyclic AMP (cAMP) concentration and activates the cAMP-dependent protein kinase A (PKA), thus causing inhibition of platelet aggregation as well as inducing peripheral vasodilation. In addition, CSZ prevents oxidative stress (Kurtoglu et al., 2014), promotes neurogenesis (Tanaka et al., 2010), acts as an anti-atherogenic agent by enhancing cholesterol elimination from macrophages (Nakaya et al., 2010), inhibits inflammatory cytokine production and signaling (Jung et al., 2010), and improves systemic lymphatic function by inducing the proliferation and stabilization of lymphatic endothelial cells (Kimura et al., 2014).

CSZ is primarily used to prevent cerebral ischemia (Shinohara et al., 2010); however, it also reported slow cognitive decline in patients with mild cognitive impairment (MCI), AD, and cerebrovascular disease (CVD) (Arai and Takahashi, 2009; Sakurai et al., 2013; Taguchi et al., 2013; Ihara et al., 2014; Tai et al., 2017). While the mechanisms of cognitive preservation remain unclear, CSZ has been shown to decrease Aβ_25–35_ accumulation and to concomitantly reduce cognitive deficits in animal models of AD (Hiramatsu et al., 2010; Park et al., 2011). Using the human-derived neuroblastoma cell line SH-SY5Y cells, we recently reported that CSZ suppressed Aβ_1–42_-induced neurotoxicity *via* the inhibition of oxidative stress, as demonstrated by coincident reduced reactive oxygen species (ROS) accumulation, mitogen-activated protein kinase (MAPK)-p38 signaling, and nicotinamide adenine dinucleotide phosphate (NADPH) oxidase activity in SH-SY5Y cells (Oguchi et al., 2017).

Although fibrils have long been considered to be the primary neurodegenerative agents, recent evidence indicate that soluble oligomers initiate neuronal and synaptic dysfunctions associated with AD (oligomer hypothesis) (Selkoe and Hardy, 2016; Ono, 2018). Furthermore, different evidence suggests that a tau pathogenesis is mediated by low-molecular-weight (LMW) oligomers of Aβ, e.g., dimers and trimers (Ittner and Gotz, 2011). If this is the case, DMTs should target the neurotoxic activity of these smaller Aβ assemblies to achieve the highest efficacy. Underscoring the potential efficacy of CSZ, we recently demonstrated the inhibitory effects of CSZ on aggregation of Aβ isoforms *in vitro*, including oligomer formation (Shozawa et al., 2018).

In this review, we evaluate the therapeutic possibility of CSZ for AD pathogenesis based on clinical and basic research findings, taking account of the present situation in which no DMTs have been available and some effective combination therapy is seriously sought after.

## Protective Effects of Cilostazol on Neuronal Cells

CSZ has been known to protect various cell types from different stressors, e.g., endothelial cells from H_2_O_2_-induced oxidative stress (Ota et al., 2008), vascular smooth muscle cells from endothelin-induced vasoconstriction (Kawanabe et al., 2012), cells constituting the blood-brain barrier (BBB) from collagenase-induced stroke damage (Takagi et al., 2017), and primary cultured hepatocytes from ethanol-induced damage (Xie et al., 2018). It would be reasonable to expect that CSZ may also be neuroprotective and effective in the treatment of AD or vascular dementia.

Types of neurodegeneration that possibly cause dementia include synaptic transmission dysfunction, neuronal cell death, CREB-related loss of long-term potentiation, and so on. Researches on possible molecular mechanisms of neuroprotection by CSZ will be reviewed in the following.

As mentioned in the Introduction, CSZ has been approved in various countries as an anti-platelet agent, whose inhibition of PDE3 results in PKA activation to suppress platelet aggregation. Some study indicated that neuroprotection by CSZ was associated with the inhibition of PDE3 (Mabuchi et al., 2001), but the molecular mechanisms underlying neuroprotection remain uncertain because PDE3 is abundantly expressed in the heart and vascular smooth muscle cells, but far less in the human brain (Lakics et al., 2010). Thus, it is unlikely that CSZ-induced PDE3 inhibition in neuronal cells is the primary mechanism for improving cognitive impairments in AD. Further, in our experiments using SH-SY5Y cells, CSZ did not reverse the decrease in cAMP concentration induced by Aβ_1–42_ exposure despite a reduction in neurotoxicity (Oguchi et al., 2017). Thus, CSZ-mediated neuroprotection seems unrelated to PDE3. In addition to its action of selective PDE3 inhibition, CSZ is known to activate other serine/threonine kinase including AMP-activated protein kinase (AMPK) (Park et al., 2016). Neuronal cells treated with CSZ exhibit increased expression of phosphorylated AMPKα, causing upregulation of Aβ autophagy and decreasing intracellular Aβ accumulation (Park et al., 2016).

Another possible protective mechanism involves the modulation of NMDA signaling that is critical in synaptic transmission. Seixas da Silva et al. (2017) recently reported that NMDA receptor (NMDAR) activation mediates the reduced AMPK activity and metabolic deficits in cultured hippocampal neurons exposed to Aβ_1–42_ oligomers. CSZ suppressed the cognitive deficits caused by an NMDAR antagonist in mice (Hashimoto et al., 2010). In this case, cAMP-response element-binding protein (CREB) decrease induced by an NMDAR antagonist was counteracted by CSZ treatment and the resulting increase in CREB suppressed the cognitive deficits. CSZ seems to activate AMPK *via* Sir1 in neurons, and this in turn activates CREB (Park et al., 2016).

CSZ appears to suppress oxidative stress through multiple mechanisms. Choi et al. (2002) first reported that CSZ can ameliorate oxidative stress by scavenging hydroxyl and peroxy radicals, thus decreasing ischemic cerebral infarction. In a recent study of mice with permanent focal cerebral ischemia, CSZ suppressed oxidative stress in ischemic neurons by reducing NADPH oxidase (NOX) 2 expression, further resulting in reduced infarct volume (Shichinohe et al., 2015). Moreover, CSZ treatment in SH-SY5Y cells significantly reduced ROS generation during Aβ_1–42_ exposure by downregulating NOX activation and Nox-4 mRNA expression (Oguchi et al., 2017).

Furthermore, CSZ treatment significantly reduced the expression of the proapoptotic protein Bax and the activation of the apoptosis effector caspases, while significantly increasing the expression of the antioxidant enzyme superoxide dismutase and the antiapoptotic protein Bcl-2 (Oguchi et al., 2017). These results suggest that CSZ attenuates Aβ_1–42_-induced cytotoxicity in neuronal cells by inhibiting NOX-derived ROS production and mitochondrial damage, resulting in reduced apoptosis.

ROS generated during the early stage of Aβ aggregation also activates the p38-MAPK and JNK signaling pathways in AD brains (Zhu et al., 2002; Tabner et al., 2005). ERK1/2 is activated by neural signals associated with synaptic plasticity and cytoprotection. In the mouse hippocampus, ERK1/2 is activated in postsynaptic neurons by NMDAR activation during long-term potentiation (LTP) induction (Schmitt et al., 2005). Calmodulin-dependent kinase kinase/calmodulin kinase I activity gates extracellular-regulated kinase-dependent LTP. NMDAR activation phosphorylates (activates) ERK1/2, which subsequently regulates the various gene expressions by the CREB phosphorylation. In our recent study, CSZ elevated ERK1/2 and CREB phosphorylation in SH-SY5Y cells treated with Aβ_1–42_ (Oguchi et al., 2017). In another cell system, that is, mouse neuroblastoma Nm2a cells with overexpression of human mutated amyloid precursor protein (APP) cells, CSZ was shown to increase CREB phosphorylation (Lee et al., 2014).

Recent reports have implicated aberrant CREB signaling in cognitive and neurodegenerative disorders. The hippocampal accumulation of Aβ peptide causes synapse loss and disrupts LTP, which is critical for encoding long-term spatial, associative, emotional, and social memories, through deficient CREB signaling (Saura and Valero, 2011). Further, Qiu et al. (2016) reported that Aβ_1–42_ oligomers induce apoptosis through decreased Akt and CREB phosphorylation in PC12 cells (Qiu et al., 2016). In addition, the exposure of SH-SY5Y cells to Aβ_1–42_ decreased phosphorylated CREB, a response prevented by CSZ, and pretreatment with a MEK1/2 inhibitor significantly suppressed CSZ-stimulated CREB phosphorylation (Oguchi et al., 2017).

**Figure 1:**
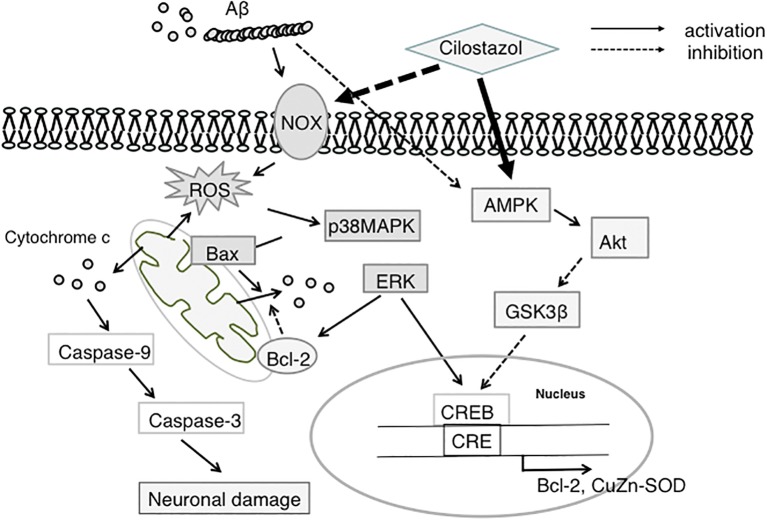
Proposed neuroprotective mechanism of CSZ against Aβ-induced neurodegeneration. This scheme shows that CSZ suppresses Aβ-induced neurotoxicity *via* ROS-activated p38MAPK and AMPK/CREB pathways. NOX, NADPH oxidase; ROS, reactive oxygen species; p38MAPK, p38 mitogen-activated protein kinase; ERK, extracellular regulated kinase; AMPK, 5′-adenosine monophosphate (AMP)-activated protein kinase; GSK3β, glycogen synthase kinase 3β; CREB, cAMP-responsive element-binding protein; SOD, superoxide dismutase.

In summary, Aβ-induced oxidative stress is inhibited by CSZ by scavenging and suppressing NOX activity. Amelioration of oxidative stress by CSZ reduces Aβ-induced activation of p38-MAPK signaling, which is strongly linked to apoptosis and inflammatory responses. Alternatively, CSZ increases ERK1/2 activity in neuronal cells, promoting CREB phosphorylation and transactivation of CRE-controlled genes including Bcl-2 (Figure 1). In addition, CSZ protects cells from mitochondrial dysfunction, which is another ROS source, by inhibiting Aβ-induced increase in Bax and activation of effector caspases. Thus, CSZ may have multiple cytoprotective actions against oxidative stress, impaired synaptic plasticity, mitochondrial dysfunction, and apoptosis and therefore can prevent neuronal damage and associated cognitive deficits in AD.

## Cilostazol Inhibits Aβ Oligomer Formation

Several studies have reported the LMW oligomers of Aβ to be particularly toxic (Shankar et al., 2007; Ono et al., 2009; Ono, 2018). LMW oligomers from APP-expressing CHO cells caused progressive dysfunction of synaptic plasticity in rat hippocampal slices (Shankar et al., 2007). Further, LMW oligomers, especially dimers, that were isolated from AD brains exerted synaptic toxicity (Shankar et al., 2008). In our combined structural and cellular studies using pure Aβ oligomers, we revealed that LMW oligomers (dimer, trimer, and tetramer) are more cytotoxic than monomer (Ono et al., 2009); this superior toxicity correlated with the increases in β-sheet content and the seeding activity to facilitate fibrillization (Ono et al., 2009).

**Figure 2:**
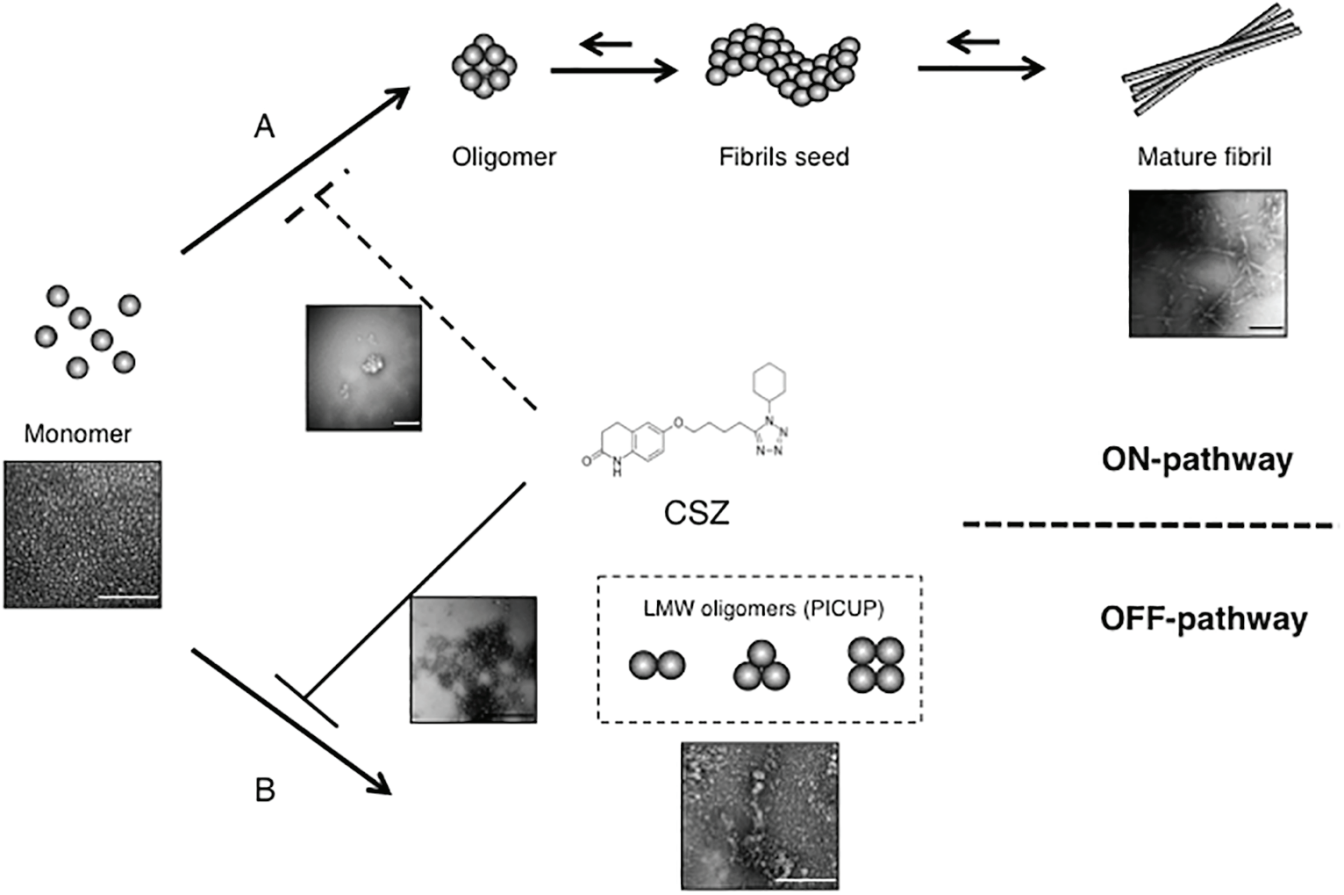
Inhibitory effects of CSZ on Aβ aggregation. The Aβ monomer may aggregate to produce toxic intermediate aggregates, such as soluble oligomers, and finally mature fibrils. CSZ inhibits ON-pathway formation of Aβ fibrils concurrently with strong prevention of OFF-pathway Aβ oligomers (scale bars = 100 nm). This research was originally published in *Neurosci. Lett.* (Shozawa et al., 2018).

Shozawa et al. (2018) recently demonstrated that CSZ significantly inhibited both Aβ_1–40_ and Aβ_1–42_ aggregation, but with a stronger inhibitory effect on oligomerization than on fibrillization. Although structural change to β-sheet and fibrillization generally correlate during peptide assembly (Levine, 1999), we reported that LMW oligomers including PICUP-derived oligomers initiated exhibiting β-sheet content at the dimer stage; conversely, a thioflavin fluorescence increase was not observed, which is indicative of fibril formation (Ono et al., 2009). Although Aβ oligomers were initially believed to be positioned on the ON-pathway from monomer to fibrils, some oligomers (e.g., amylospheroids and PICUP-derived oligomers) are positioned on the OFF-pathway but exhibit higher toxicity (Hoshi et al., 2003; Ono, 2018). Recently, we reported high-molecular-weight oligomers, e.g., protofibrils to also be positioned on the OFF-pathway using combined thioflavin T assay, electron microscopy, and high-speed atomic force microscopy (Watanabe-Nakayama et al., 2016). Thus, an explanation for the superior inhibitory potency of CSZ against Aβ oligomerization than against fibrillization is the fact that LMW oligomers generated by PICUP are positioned on the OFF-pathway (Figure 2; Shozawa et al., 2018).

Until now, we reported that several hydroxyl radical scavengers, e.g., rosmarinic acid, curcumin, and rifampicin, exhibit inhibitory effects on Aβ, tau, and α-synuclein (αS) oligomer formation (Ono et al., 2012; Takahashi et al., 2015; Umeda et al., 2016). Yen and Hsieh (1997) and Tomiyama et al. (1996) reported a phenolic compound with hydroxyl groups, particularly orthoquinone and naphthohydroquinone to be a good hydroxyl radical scavenger. Based on binding assays, we hypothesized that the orthoquinone ring of rosmarinic acid and curcumin and naphthohydroquinone of rifampicin facilitate their specific binding to free Aβ/tau/αS, thereby inhibiting aggregation (Takahashi et al., 2015; Umeda et al., 2016). Regardless of the absence of a quinone ring in CSZ, its quinolone ring with free radical scavenging activity may be associated with Aβ binding and/or inhibition of Aβ oligomerization (Figure 2; Orhan Puskullu et al., 2013; Shozawa et al., 2018).

CSZ reportedly suppresses Aβ accumulation-induced tauopathy *via* increased PKA-linked CK2/SIRT1 expression *in vitro* (Lee et al., 2014). Additionally, the oral administration of CSZ to C57BL/6J mice prior to Aβ_25–35_ injection showed significant improvement in spatial learning and memory, prevented Aβ-induced immunoreactivity of Aβ and phosphorylated tau, and suppressed microglia activation compared with control Aβ_25–35_-injected mice. Nevertheless, post-treatment with CSZ following Aβ_25–35_ administration and Aβ accumulation did not reduce Aβ-induced neuropathology. Moreover, CSZ had no effect on neprilysin and insulin-degrading enzyme involved in Aβ peptide degradation (Park et al., 2011). Additionally, in a mouse model of cerebral amyloid angiopathy (CAA), CSZ facilitated soluble Aβ clearance and rescued cognitive deficits (Maki et al., 2014).

Very recently, administration of CSZ was reported to increase proteasome activity and reduce the levels of total and aggregated tau species and cognitive decline in a mouse model of tauopathy (Schaler and Myeku, 2018).

In summary, these findings suggest that CSZ promotes the clearance of Aβ oligomers and blocks Aβ oligomer formation, thereby preventing tau pathogenesis. On the other hand, it does not facilitate the clearance of mature fibrils, possibly limiting its clinical efficacy in advanced AD.

## Cilostazol Improves Cognitive Decline in Patients with Alzheimer’s Disease

As an antiplatelet therapy, patients generally use 100 mg CSZ orally twice daily; hence, one potential mechanism for cognitive improvement is anti-thrombotic activity and prevention of focal ischemia. At this dose, the plasma concentration attains a steady state between 1.5 and 3.2 μM. Similarly, rats orally administered 10 mg/kg CSZ had a plasma concentration of 993 ng/ml (2.69 μM) as measured by radioactive carbon; however, the concentration was only 99 ng/g in the cerebrum and 946 ng/g in the hypophysis following an oral administration of 10 mg/kg CSZ, suggesting only a minor fraction of CSZ passes through the BBB (Akiyama et al., 1985). Whether the prevention of Aβ oligomer formation, neurodegeneration, and cognitive impairment can occur in patients with AD at these clinical CSZ doses needs to be clarified.

The concentrations required to prevent Aβ aggregation are 10- to 40-fold higher (25–100 μM) than the effective concentration of 2.5 μM identified in the present Aβ toxicity assay, which is notably within the range of normal plasma concentrations. Further, its brain concentrations may be substantially lower than its plasma concentrations. However, the cerebrospinal fluid concentrations of Aβ were only 200–300 pg/ml (~50 pM) in patients with AD (Hu et al., 2015), which is approximately 1,000,000-fold lower than the Aβ concentrations observed in this aggregation study. Considering the effective Aβ to CSZ ratio, it needs to examine whether a long-term clinical administration of CSZ continues to inhibit Aβ oligomer formation *in vivo*.

In Japan and other Asian countries, CSZ is clinically used to prevent cerebral ischemic diseases (Shinohara et al., 2010), including CAA, because it carries a limited risk of hemorrhage in most elderly patients (Charidimou et al., 2012; Saito and Ihara, 2014). The second CSZ Stroke Prevention Study (CSPS2) for patients with cerebral infarction reported hemorrhagic stroke to be significantly less frequent in a CSZ group than in an aspirin group (Shinohara et al., 2010; Uchiyama et al., 2014). These effects may be explained, at least partially, by an inhibitory effect on matrix metalloproteinase-9 expression and the protection of vascular endothelial cells (Hase et al., 2012; Kasahara et al., 2012).

The efficacy of CSZ in patients with MCI (Taguchi et al., 2013), AChEI-treated patients with clinically probable AD (Arai and Takahashi, 2009; Tai et al., 2017), and patients with AD and CVD (Sakurai et al., 2013; Hishikawa et al., 2017) has been evaluated in several small-scale clinical studies. In a pilot study involving 10 patients with moderate AD who were administered AChEI donepezil, a 5- to 6-month add-on CSZ treatment significantly increased the Mini Mental State Examination score in comparison with the baseline score (Arai and Takahashi, 2009). In a larger pilot study comprising 30 participants, a 12-month CSZ add-on therapy improved cognitive impairments in those with stable AD (Tai et al., 2017). Recently, a pilot study including 101 patients with AD and asymptomatic lacunar infarction reported that combination therapy with CSZ and the AChEI galantamine significantly improved the Geriatric Depression Scale and Abe’s behavioral and psychological symptoms of dementia scores and a 6-month CSZ monotherapy significantly improved the Geriatric Depression Scale score (Hishikawa et al., 2017). The effects of a 6-month CSZ treatment on cognition and regional cerebral blood flow (rCBF) were examined in 20 elderly patients with mild-to-moderate AD and CVD (Sakurai et al., 2013). As the results showed, the CSZ group did not show any changes in cognitive function, whereas the control group showed a cognitive decline on the AD Assessment Scale-Cognitive Subscale. Analysis of treatment effect revealed that the CSZ group showed increased rCBF in the right anterior cingulate lobe, whereas the control group showed decreased rCBF in the left middle temporal gyrus. On the other hand, initiated study in 2011 by the Seoul National University Hospital revealed that no difference between CSZ and placebo groups was reported on cognitive measures, which included the MMSE and the cognitive scale of the cognitive part of the AD Assessment Scale in 36 mild-to-moderate AD patients with subcortical white matter hyperintensities treated with donepezil for a 6-month period (Prickaerts et al., 2017).

Furthermore, an approximately 2-year retrospective analysis concluded that CSZ improves cognitive function in patients with MCI (Taguchi et al., 2013). Randomized placebo-controlled clinical phase II trials are currently ongoing for patients with MCI (Saito and Ihara, 2014).

Side effects of CSZ include most commonly headache, diarrhea, abnormal stools, irregular heart rate, and palpitations. It is contraindicated in patients with severe heart failure or severe hepatic/renal impairment (Chapman and Goa, 2003).

## Conclusion

CSZ was reported to promote Aβ clearance, inhibit Aβ oligomerization, and suppress Aβ-induced neurotoxicity *in vitro* and *in vivo*. CSZ is reported to suppress cognitive decline progression in some patients with MCI or AD. For examination of these effects in a larger scale, randomized placebo-controlled phase II trials are ongoing for patients with MCI (Saito and Ihara, 2014). As future direction, potential effects of CSZ on AD comorbidities, such as depression or metabolic dysfunctions (e.g., diabetes), will also have to be examined in AD or MCI patients with or without CVD because oxidative stress plays the important role in these diseases as in AD (Novais and Starkstein, 2015; Karki et al., 2017; Morgese et al., 2017).

## Author Contributions

KO and MT wrote the manuscript and approved the final version of the manuscript.

### Conflict of Interest Statement

The authors declare that the research was conducted in the absence of any commercial or financial relationships that could be construed as a potential conflict of interest.
